# Differences by race and ethnicity in drug use patterns, harm reduction practices and barriers to treatment among people who use drugs in Rhode Island

**DOI:** 10.1186/s12954-025-01191-2

**Published:** 2025-03-20

**Authors:** Samantha Parker, Nya Reichley, Katie B. Biello, Jacqueline Goldman, Jane A. Buxton, Scott E. Hadland, Susan G. Sherman, Brandon D.L. Marshall, Alexandria Macmadu

**Affiliations:** 1 Department of Epidemiology, Brown University School of Public Health, 121 South Main Street, Box G-S-121-2 Providence, Providence, RI 02912 USA; 2 Rhode Island Department of Health, Providence, RI USA; 3 Department of Behavioral and Social Sciences, Brown University School of Public Health, Providence, RI USA; 4 Center for Health Promotion and Health Equity, Brown University School of Public Health Providence, Providence, RI USA; 5 The Fenway Institute, Fenway Health, Boston, MA USA; 6 School of Population and Public Health, University of British Columbia, Vancouver, BC Canada; 7 Department of Pediatrics, Harvard Medical School, Boston, MA USA; 8 Department of Health, Behavior, and Society, Johns Hopkins Bloomberg School of Public Health, Baltimore, MD USA

**Keywords:** Overdose prevention, Harm reduction

## Abstract

**Background:**

As in much of the United States, there have been significant increases in overdose deaths among non-Hispanic Black and Hispanic/Latinx populations in Rhode Island over the past decade. Given the shifting dynamics of the overdose epidemic, there is an urgent need for focused interventions that address the specific needs of diverse communities. This study explores differences in drug use patterns, harm reduction behaviors and types and barriers to treatment by race and ethnicity.

**Methods:**

This study utilized baseline data from the Rhode Island Prescription and Illicit Drug Study (RAPIDS). We assessed sociodemographic characteristics, drug use patterns, harm reduction practices, treatment type, and barriers to treatment in a cross-sectional analysis of people who use drugs (PWUD), stratified by race and ethnicity (non-Hispanic white, non-Hispanic Black, non-Hispanic other race, and Hispanic). Chi-square tests of independence and ANOVA tests were used to identify statistically significant differences by race and ethnicity.

**Results:**

Among 509 participants, the median age was 43, and the majority were men (64%). Non-Hispanic Black participants reported significantly less regular use of unregulated opioids, such as heroin (10%) and fentanyl (12%), as compared to non-Hispanic white participants (39% and 33%, respectively). Non-Hispanic Black participants reported significantly less experience responding to overdoses: only 39% had ever administered naloxone and 34% had ever performed rescue breathing, as compared to 67% and 57% among non-Hispanic white participants, respectively. Despite significant differences in drug use patterns, there were few differences in harm reduction practices by race and ethnicity. Current treatment enrollment was highest among those who were non-Hispanic white (38%) and lowest among those who were non-Hispanic Black (7%).

**Conclusions:**

These findings suggest that there are differences in overdose response experience and treatment exposure between non-Hispanic Black PWUD and those belonging to other racial and ethnic groups, indicating a need for enhanced investment in overdose response education, naloxone distribution and treatment access for non-Hispanic Black PWUD.

## Introduction

The overdose epidemic in the United States is a worsening and evolving public health crisis, characterized by four distinct waves. Initially propelled by deaths involving prescription opioids, the epidemic later saw a surge in heroin-related overdoses, followed by a devastating wave fueled by synthetic opioids like fentanyl [1, 2]. The latest wave has been driven by polysubstance use and the involvement of stimulants (such as powder and crack cocaine and methamphetamine) in overdose deaths [3, 4]. While the overdose crisis has been framed as a “white problem” over much of the past two decades, recent data have documented significant increases in overdose deaths among non-Hispanic Black and Hispanic populations [5].

Both nationally and in Rhode Island, surveillance systems have documented significant increases in overdose deaths among non-Hispanic Black and Hispanic populations [6, 7]. In 2020, the national opioid overdose death rate among Black Americans was higher than that of white Americans for the first time since 1999 (36.8 vs. 31.6 overdose deaths per 100,000 people) [5]. In 2023, overdose death rates in Rhode Island were 5% higher among Hispanic/Latinx state residents as compared to white residents (37.5 vs. 35.6 overdose deaths per 100,000 people) and 43% higher among Black residents (55.2 per 100,000 people) [6]. These disparities are not merely statistical; they are rooted in systemic racism that permeates structural and social determinants of health, resulting in unequal access to opioid use disorder treatment and harm reduction services [8]. As such, it is vital to identify opportunities for focused interventions that address the specific needs of diverse communities [9].

To this end, the objectives of this analysis were to: (1) explore variations in drug use patterns and harm reduction practices by race and ethnicity in a community-recruited sample of people who use drugs (PWUD) in Rhode Island, and (2) to characterize substance use disorder (SUD) treatment history and barriers to accessing treatment by race and ethnicity. Our examination of differences in drug use patterns and harm reduction practices was exploratory, and we hypothesized that non-Hispanic white PWUD would have greater access and engagement in substance use treatment as compared to Black and Hispanic PWUD due to differences in social determinants and structural barriers.

## Methods

### Study design and data sources

This study utilized baseline data from the Rhode Island Prescription and Illicit Drug Study (RAPIDS), a clinical trial assessing the efficacy of a fentanyl test strip intervention on self-reported overdose among a cohort of PWUD in Rhode Island. The study protocol and procedures have been described in detail previously [10]. In brief, we enrolled 509 people who use drugs between August 2020 and February 2023. To be eligible for the study, participants had to be: (1) currently residing in the state of Rhode Island; (2) between the ages of 18 and 65; (3) able to complete a survey in English; and (4) use diverted or counterfeit prescription pills, crack cocaine, powder cocaine, crystal methamphetamine, heroin and/or fentanyl or reported any injection drug use in the 30 days prior to screening. Participants were identified through field-based recruitment, statewide public transportation advertising and internet-based advertisement, and directly from syringe services programs.

RAPIDS participants completed study assessments at baseline, 1-, 2-, 3-, 6- and 12-months post-baseline. The present study utilizes data from the baseline questionnaire, which was completed by all participants and assessed sociodemographic characteristics, drug use patterns, harm reduction practices and various clinical characteristics, including engagement in substance use treatment. Participants received $35 USD for completing the baseline visit. All study procedures were approved by the Brown University Institutional Review Board.

### Key variables

The primary independent variable in this study was self-reported race and ethnicity. The study utilized a mutually exclusive, four-category race and ethnicity variable that was derived from participants’ self-reported race and ethnicity: (1) non-Hispanic white (2), non-Hispanic Black (3), Hispanic/Latinx of any race and (4) non-Hispanic other race, which included any persons identifying as American Indian or Alaska Native, Asian, Native Hawaiian or Pacific Islander or as mixed, bi-racial or multi-racial. Hispanic ethnicity was derived from participants’ response to the item “Are you of Hispanic or Latino descent?” (yes vs. no). Persons whose race was categorized as “non-Hispanic Black” included those self-identified as having African, Haitian, or Cape Verdean ancestry. Additionally, participants who responded “other” were reviewed for self-reported answers. Some participants who responded “other” self-identified as “Hispanic” (n = 9), “Puerto Rican” (n = 7), “Latino” (n = 4), or Spanish (n = 4) in their written response; these respondents were classified as Hispanic/Latinx of any race. In this paper, racial and ethnic categories are capitalized except for ‘white’ to acknowledge its historical treatment as an unmarked default. This follows conventions in critical race scholarship and style guides such as AP.

There were three primary categories of dependent variables examined by this study: (1) individual sociodemographic characteristics; (2) drug use patterns and harm reduction practices; and (3) types of and barriers to SUD treatment. Sociodemographic characteristics included age (in years), current gender identity (cisgender man, cisgender woman, transgender/other), sexual orientation (LGBQ vs. heterosexual), lifetime homelessness (yes vs. no), monthly income from employment or government sources (≤$500, $501–1500, >$1500), and lifetime incarceration (yes vs. no). Measures of sociodemographic characteristics and drug use patterns were pilot tested in our earlier work [11].

Drug use patterns examined included: nonmedical regular use (i.e., at least 4 days of use in the prior month; approximately “at least weekly” use, consistent with our prior work [12–14]) of prescription opioids (yes vs. no) and benzodiazepines (yes vs. no); and regular use of methamphetamine (yes vs. no), powder cocaine (yes vs. no), crack cocaine (yes vs. no), and heroin/fentanyl (yes vs. no). Drug use frequency was assessed using a modified risk behavior assessment, which has been found to be both reliable and valid in extensive earlier research [15, 16]. Additional drug use patterns examined were: lifetime injection drug use (yes vs. no), lifetime experience with sharing syringes or other injecting supplies (yes vs. no), lifetime drug selling (yes vs. no), ever experiencing an overdose (yes vs. no), prior month overdose history (yes vs. no), and number of prior month overdoses (median). Overdose response experiences were examined, including ever witnessing an overdose (yes vs. no), ever performing rescue breathing (yes vs. no), and ever administering naloxone (yes vs. no). Current harm reduction practices included the following: avoid mixing with alcohol (yes vs. no), avoid mixing with other drugs (yes vs. no), smell or taste my supply (yes vs. no), using with someone else (yes vs. no), take smaller amounts (yes vs. no), go slow (yes vs. no), take a tester (yes vs. no), use fentanyl test strips (yes vs. no), keep naloxone nearby (yes vs. no), change drug supplier (yes vs. no), or nothing (yes vs. no); for each variable except for “nothing”, “yes” corresponds with the protective practice.

Substance use treatment characteristics examined were: any lifetime treatment (yes vs. no), any current treatment (yes vs. no) and type of current treatment (opioid agonist therapy vs. another form of treatment). Lifetime and current treatment were inclusive to detox, self help groups (e.g., 12-step, AA, NA), outpatient, partial hospitalization, residential, and other program types. We also assessed any lifetime experience of being unable to access treatment (yes vs. no), and among those, we assessed types of barriers to access. Treatment barriers included: could not afford (yes vs. no), waiting list (yes vs. no), not allowed by health insurance (yes vs. no), did not know of any programs (yes vs. no), programs weren’t youth friendly (yes vs. no), turned down by a program (yes vs. no), no nearby program (yes vs. no), no beds available (yes vs. no), or another reason (yes vs. no).

### Statistical analyses

R version 4.3.2 was used for all statistical analyses. Medians (for continuous variables) and proportions (for categorical variables) are presented for all measures for the overall sample and stratified by non-Hispanic white, Non-Hispanic Black, Hispanic and non-Hispanic other races. Chi-square tests of independence were utilized to assess whether differences in distribution were significant between the four racial and ethnic groups for categorical variables. ANOVA tests were employed to test for differences in means among the four racial and ethnic groups for continuous variables (i.e., age and number of overdoses in the prior month). For both variables, we confirmed that key assumptions for ANOVA tests (e.g., homogeneity of variance) were met to ensure that this test was appropriate.

## Results

Among 509 PWUD, the median age was 43 (interquartile range [IQR]: 35, 53), and the majority were cisgender men (*n* = 326, 64%; see Table 1). Of the 509 participants, 51% were non-Hispanic white (*n* = 261), 16% were non-Hispanic Black (*n* = 82), 12% were non = Hispanic other race (*n* = 59) and 21% were Hispanic/Latinx (*n* = 106). Sociodemographic characteristics were broadly similar across racial and ethnic groups, except that non-Hispanic Black participants were somewhat older (median age 51; IQR: 41, 56). Most participants had any lifetime experience with incarceration (*n* = 394, 77%), and most reported experiencing homelessness in the prior month (*n* = 299, 59%).

**Table 1:** Sociodemographics, drug use patterns, and harm reduction practices of 509 people who use drugs in Rhode Island from August 2020 to February 2023, stratified by race and ethnicity

Characteristic		Overall (*n* = 509)	Non-Hispanic white (*n* = 261)	Non-Hispanic Black^a^ (*n* = 82)	Non-Hispanic other race (*n* = 59)	Hispanic (*n* = 106)	*P*-value
***Sociodemographics***							
Age	By year, median (IQR)	43 (35, 53)	42 (35, 52)	51 (41, 56)	40 (35, 52)	39 (31, 51)	< 0.01
Gender	Man (cisgender)	326 (64%)	166 (63%)	51 (61%)	32 (54%)	77 (73%)	0.11
	Woman (cisgender)	166 (33%)	88 (34%)	30 (36%)	22 (37.3%)	26 (25%)	- - -
	Transgender/Other^a^	17 (3%)	7 (3%)	2 (2%)	5 (9%)	3 (3%)	- - -
Orientation	LGBQ	83 (16%)	41 (15%)	12 (15%)	13 (22%)	17 (16%)	0.64
Homelessness	Prior month	299 (59%)	146 (56%)	44 (53%)	39 (66%)	70 (66%)	0.13
Monthly income	≤$500	227 (45%)	117 (45%)	39 (47%)	19 (32%)	52 (49%)	0.14
	$501–1500	236 (47%)	114 (44%)	41 (49%)	35 (59%)	46 (43%)	- - -
	<$1500	41 (8%)	27 (10%)	3 (4%)	4 (7%)	7 (7%)	- - -
Incarceration	Ever	394 (77%)	205 (79%)	64 (77%)	47 (80%)	78 (74%)	0.73
***Drug use patterns***							
Regular use	Prescription opioids^b^	124 (24%)	68 (26%)	16 (19%)	8 (14%)	32 (30%)	0.06
	Benzodiazepines^b^	112 (22%)	74 (28%)	12 (14%)	8 (14%)	18 (17%)	< 0.01
	Methamphetamine^b^	83 (16%)	59 (23%)	2 (2%)	9 (15%)	13 (12%)	< 0.01
	Powder cocaine^b^	137 (27%)	56 (22%)	27 (33%)	17 (28%)	37 (35%)	0.03
	Crack cocaine^b^	309 (61%)	155 (59%)	51 (61%)	33 (56%)	70 (66%)	0.56
	Heroin/Fentanyl^c^	200 (39%)	126 (48%)	12 (15%)	19 (32%)	43 (41%)	< 0.01
Injection drug use	Ever	280 (55%)	183 (70%)	20 (24%)	28 (48%)	49 (46%)	< 0.01
Share syringes/supplies	Ever	169 (33%)	122 (47%)	6 (7%)	20 (34%)	21 (20%)	< 0.01
Drug selling	Ever	349 (69%)	188 (72%)	48 (58%)	41 (70%)	72 (68%)	0.11
Overdose history	Ever	273 (54%)	163 (63%)	26 (31%)	29 (49%)	55 (52%)	< 0.01
	Prior month	39 (8%)	20 (8%)	3 (4%)	4 (7%)	12 (11%)	0.26
	# prior month overdoses (median, IQR)	1 (1,2)	2 (1,3)	1 (1,1)	3 (1.75, 3.75)	1.5 (1, 2)	0.30
Overdose response	Ever witness	434 (85%)	227 (87%)	61 (74%)	52 (88%)	94 (89%)	0.01
	Ever perform rescue breathing	260 (51%)	149 (57%)	28 (34%)	29 (49%)	54 (51%)	< 0.01
	Ever administer naloxone	307 (60%)	175 (67%)	32 (39%)	37 (63%)	63 (59%)	< 0.01
***Harm reduction practices***	Avoid mixing with alcohol	113 (22%)	67 (26%)	11 (13%)	8 (14%)	27 (26%)	0.03
	Avoid mixing with other drugs	144 (28%)	75 (29%)	18 (22%)	12 (20%)	39 (37%)	0.06
	Smell or taste my supply	96 (19%)	53 (20%)	11 (13%)	11 (19%)	21 (20%)	0.55
	Using with someone else	169 (33%)	102 (39%)	17 (21%)	15 (25%)	35 (33%)	< 0.01
	Take smaller amounts	115 (23%)	113 (43%)	35 (42%)	26 (44%)	49 (46%)	0.95
	Go slow	201 (40%)	95 (36%)	30 (36%)	33 (56%)	50 (47%)	0.21
	Take a tester	115 (23%)	57 (22%)	17 (21%)	13 (22%)	28 (26%)	0.75
	Use fentanyl test strips	90 (17%)	47 (18%)	10 (12%)	13 (22%)	20 (19%)	0.44
	Keep naloxone nearby	219 (43%)	121 (46%)	26 (31%)	25 (42%)	47 (44%)	0.12
	Change drug supplier	87 (17%)	38 (15%)	16 (19%)	12 (20%)	21 (20%)	0.48
	Something else^d^	66 (13%)	35 (13%)	11 (13%)	9 (15%)	11 (10%)	0.94
	Nothing	38 (8%)	21 (8%)	5 (6%)	4 (7%)	8 (8%)	0.81

^a^ Includes self-identified African, Haitian, and Cape Verdean ancestry

^b^ Indicates at least 4 days of use in the prior 30 days

^c^ Indicates “at least weekly” or “every day” use frequency

^d^ Includes the following other participant-generated responses: Stop or avoid drug use; avoid drugs with overdose risk (e.g., heroin, fentanyl); use same drug supplier or talk to supplier, cook or re-cook drugs; use responsibly; drink water; know body limits; pay attention to signs of overdose (nodding off); visually inspect drugs for fentanyl; move around or more than usual; stick to usual amounts/routine

We identified significant differences in drug use patterns by race and ethnicity. Non-Hispanic Black participants rarely reported regular use of methamphetamine (*n* = 2, 2%) as compared to other racial and ethnic groups (e.g., 23% among non-Hispanic white participants), and nonmedical, regular use of benzodiazepines was significantly higher among non-Hispanic white participants (*n* = 74, 28%) as compared to other racial and ethnic groups (e.g., 14% among non-Hispanic Black participants). Additionally, non-Hispanic Black participants reported significantly less regular use of heroin/fentanyl, 15% (*n* = 12) as compared to other racial and ethnic groups (e.g. 48% and 41% among non-Hispanic white and Hispanic participants, respectively). Notably, we did not identify any significant differences in crack cocaine use by race and ethnicity (*p* = 0.56) or in lifetime drug selling (*p* = 0.11), although non-Hispanic white participants reported greater lifetime experience with injecting drugs (*n* = 183, 70%), sharing syringes or supplies (*n* = 122, 47%), and overdose (*n* = 163, 63%) as compared to other racial and ethnic groups (e.g., 24%, 7%, and 31%, respectively, among non-Hispanic Black participants). Non-Hispanic Black participants had significantly fewer experiences responding to overdose: fewer non-Hispanic Black participants reported ever administering naloxone (*n* = 32, 39%) or ever having performed rescue breathing (*n* = 28, 34%) as compared to other racial and ethnic groups (e.g., 57% among non-Hispanic white participants), although non-Hispanic Black participants were also less likely to report ever witnessing an overdose as compared to other racial and ethnic groups (*p* = 0.01).

Despite significant differences in drug use patterns, there were few differences in harm reduction practices by race and ethnicity, although significantly fewer non-Hispanic Black (*n* = 17, 21%) and non-Hispanic participants of other races (*n* = 15, 25%) reported using with someone else as compared to non-Hispanic white participants (*n* = 102, 39%).

Experiences with SUD treatment, stratified by race and ethnicity, are presented in Table 2. Any lifetime experience with treatment was highest among non-Hispanic white (*n* = 225, 86%) and non-Hispanic other race participants (*n* = 53, 90%) and lowest among non-Hispanic Black participants (*n* = 57, 69%). Current treatment enrollment was also highest among those who were non-Hispanic white (*n* = 99, 38%) and lowest among those who were non-Hispanic Black (*n* = 6, 7%). Those who were non-Hispanic Black rarely reported any current engagement with opioid agonist therapy (*n* = 2, 2%), while more than a third of non-Hispanic white participants reported current engagement with opioid agonist therapy (*n* = 89, 34%). Though there were significant differences in lifetime experience with being unable to access treatment (*p* = 0.03), there was very little variation in barriers when we stratified by race and ethnicity.

**Table 2:** Experiences with substance use treatment among 509 people who use drugs in Rhode Island who were unable to access treatment, stratified by race and ethnicity

Characteristic		Overall (*n* = 509)	Non-Hispanic white (*n* = 261)	Non-Hispanic Black^a^ (*n* = 82)	Non-Hispanic other race (*n* = 59)	Hispanic (*n* = 106)	*P* - value
Any lifetime treatment	Yes	415 (82%)	225 (86%)	57 (69%)	53 (90%)	80 (76%)	< 0.01
Any current treatment	Yes	143 (28%)	99 (38%)	6 (7%)	24 (23%)	14 (24%)	< 0.01
Type of current treatment	Opioid agonist therapy ^b^	125 (25%)	89 (34%)	2 (2%)	12 (20%)	22 (21%)	< 0.01
	Other ^c^	29 (6%)	20 (8%)	4 (5%)	2 (3%)	3 (3%)	0.24
Ever unable to access treatment	Yes	207 (41%)	117 (45%)	22 (27%)	42 (40%)	26 (44%)	0.03
Type of barrier to access (*n* = 207)^d^	Could not afford or no health insurance	24 (5%)	17 (15%)	1 (5%)	2 (7%)	4 (10%)	0.58
	Waiting list	48 (23%)	24 (21%)	25 (23%)	35 (17%)	10 (24%)	0.50
	Not allowed by health insurance	35 (17%)	22 (19%)	5 (23%)	5 (19%)	3 (7%)	0.29
	Turned down by a program	37 (18%)	25 (21%)	4 (18%)	5 (19%)	3 (7%)	0.23
	No beds available	84 (40%)	42 (36%)	7 (32%)	13 (50%)	22 (52%)	0.16
	Another reason ^e^	67 (32%)	37 (32%)	3 (13%)	18 (42%)	9 (35%)	0.11

^a^ Includes self-identified African, Haitian, and Cape Verdean ancestry

^b^ Includes methadone and Buprenorphine, Suboxone and Subtex treatment

^c^ Includes detox (*n* = 1), self-help group (*n* = 9), outpatient treatment (*n* = 15), residential treatment (*n* = 8), and other not specified treatment (*n* = 6)

^d^ Barriers are not mutually exclusive

^e^ Includes “Did not know of any programs,” “Programs weren’t youth-friendly,” and “No nearby program” and the following other participant-generated responses: Covid, did not meet drug use criteria, barriers related to transportation, lack of identification or correct paperwork, program didn’t accept transgender individuals, was not ready to enter treatment, couldn’t continue to use other substances like marijuana or alcohol in treatment, detox before treatment

## Discussion

In this study, we sought to examine racial and ethnic differences in drug use patterns, harm reduction practices and barriers to treatment in a cohort of people who use drugs in Rhode Island. There were identifiable differences in drug use patterns; for example, non-Hispanic Black participants reported significantly less regular use of methamphetamines and heroin/fentanyl compared to non-Hispanic white participants. Additionally, we found that non-Hispanic Black participants had fewer overdose response experiences, reporting lower rates of ever performing rescue breathing or administering naloxone compared to the other racial and ethnic groups. Finally, we found that a significantly lower percentage of non-Hispanic Black participants reported lifetime or current enrollment in substance use treatment compared to non-Hispanic white participants.

In reporting these findings, we emphasize that racial and ethnic differences are the result of systemic racism, and unequal access to healthcare, treatment, and the social drivers of health [8]. To our knowledge, this study is among the first to comprehensively explore variations in drug use patterns, harm reduction behaviors, and types and barriers to treatment by race and ethnicity among people who are using drugs in the current fourth wave of the overdose crisis. While one recent study from a team at the Rhode Island Department of Health examined variations in substance use and harm reduction practices by race and ethnicity in a similar population of PWUD [17], the current study confirms and extends this work by leveraging a larger sample, employing measures of drug use frequency, exploring a wider variety of harm reduction practices, and critically, by considering differences in experiences with substance use treatment and barriers to treatment by race and ethnicity, which is a novel contribution of the present work.

We identified several significant differences in drug use patterns across the racial and ethnic groups examined. Black participants in our study reported notably less regular use of methamphetamine and heroin/fentanyl as compared to their white counterparts. These findings are consistent with those reported by Rodriguez et al. indicating that Black participants had significantly lower reported regular use of methamphetamine and fentanyl as compared to participants from other racial and ethnic groups. However, while our study found that Black participants reported significantly less regular use of benzodiazepines than their white counterparts, Rodriguez et al., 2023 did not observe significant differences in benzodiazepine use within the population studied [17], This may be explained by the fact that Rodriguez et al. stratified benzodiazepine consumption by prescription and non-prescription use. Moreover, some racial and ethnic differences in drug use patterns may reflect cultural, geographic, or social factors (e.g., more regular methamphetamine use among white participants), whereas other differences may reflect historical trends driven by systemic inequities (e.g., less regular use of heroin/fentanyl among Black participants stemming from racialized differences in opioid prescribing and opioid exposure). Understanding these dynamics—as well as the historical contexts in which they are embedded—is crucial for developing tailored and responsive interventions that address health inequities across racial and ethnic groups.

Historically, regular crack cocaine use has been attributed to communities of color, particularly Black PWUD [5]. However, similar to Rodriguez et al., we identified no significant difference in regular crack cocaine use across the four racial and ethnic groups, even as crack cocaine was the most commonly reported drug used regularly across all racial/ethnic groups (61% of the overall sample). This may imply a contemporary shift in drug use patterns toward more widespread use of crack cocaine, which is broadly consistent with literature describing the fourth wave of the epidemic, characterized by polysubstance use and increasing overdoses attributable to psychostimulants. Overall, current findings underscore the need for increased investment in funding evidence-based treatments for stimulant use disorder, as well as a need to tailor harm reduction outreach efforts to the needs of PWUD who are engaged in stimulant and polysubstance use [18, 19]. For example, dispensation of naloxone to PWUD regardless of primary drug(s) of choice and enhanced distribution of safer smoking kits are both promising strategies to engage PWUD who use stimulants [20].

Despite some variation in drug use patterns, we found few differences in harm reduction practices across the four racial and ethnic groups. Of note, we found that Black PWUD were significantly less likely to describe employing “using with someone else” as a harm reduction practice as compared to other racial and ethnic groups. Solitary drug use has long been known to be a risk factor for fatal overdose both in Rhode Island and nationally [21, 22]. As such, these findings underscore the need for increased investment in overdose prevention centers (OPCs), which are spaces where PWUD can use pre-obtained drugs under trained supervision. In 2021, New York City became the first setting in the United States to open and operate publicly recognized OPCs [23], and Rhode Island recently became the first state to legislatively authorize OPCs, which are expected to begin operating in fall 2024 [24]. Smoking rooms are available at OPCs in New York City, and in Rhode Island, facilities are required by statute to support supervised inhalation [25]. Our finding that Black, Hispanic PWUD and PWUD of other races are less likely to inject drugs reinforces the need for supervised inhalation services in OPCs in the United States to ensure racial equity in our ongoing response to the overdose crisis.

This study found that Black participants were significantly less likely to have experience with administering naloxone and performing rescue breathing. While less overdose response experience may be attributable, in part, to our finding that Black participants were less likely to report ever witnessing an overdose, this large difference likely cannot be fully explained by differences in opportunities to respond to an overdose and indicates a need for enhanced access to overdose education and naloxone distribution in communities of color. In Rhode Island, Rodriguez et al. found that Black PWUD were significantly less likely to access naloxone as a harm reduction measure compared to PWUD of other races [17]. A study from New York City found significant gaps in the training and distribution of naloxone between Black and white PWUD, with Black PWUD being less likely to be trained to administer naloxone and less likely to possess naloxone [26]. Additionally, a recent study from Massachusetts found that non-Hispanic white people received naloxone from harm reduction initiatives at higher rates per opioid-related overdose death than other racial and ethnic minority groups [27]. Given the increasing burden of overdose deaths within communities of color, our findings indicate a distinct need to invest in overdose education and naloxone distribution and outreach to Black PWUD regardless of substances used and mode of use. Further, additional research is needed to discern within-group variation among non-Hispanic Black and Hispanic populations, given that these groups are themselves heterogeneous. In the present study, non-Hispanic Black categorization was inclusive to self-identified African, Haitian, and Cape Verdean ancestry, and recent research examining Hispanic overdose decedents in Rhode Island identified diverse countries of origin, including the Dominican Republic, Guatemala, Puerto Rico, Colombia, and El Salvador [28].

Lastly, we found that both lifetime and current enrollment in substance use treatment was significantly lower among Black PWUD as compared to other racial and ethnic groups. Until the mid-2010s, non-Hispanic white people had the highest rates of opioid-related overdose mortality, and the overdose crisis was framed as an epidemic whose predominant impact was in white communities [5, 29–31]. As such, early treatment expansion efforts focused on white communities [29, 32] and the legacy of these racially imbalanced resource allocations appears to be reflected in the current sample. In the present study, we found that just 2% of Black PWUD reported current enrollment in opioid agonist therapy. While regular use of unregulated opioids was lower among Black PWUD, this level of engagement in opioid agonist therapy appears to be somewhat lower than would be expected based on these differences alone. Findings from our study are consistent with other research that non-Hispanic Black PWUD have lower engagement with substance use treatment [33, 34]. Previous research has documented that the racial and ethnic composition of a community is associated with the type of opioid agonist therapy that is locally accessible to people with opioid use disorder, with white communities having more facilities providing buprenorphine per capita and communities of color having more methadone clinics per capita [35]. Additionally, Black PWUD are more likely not to complete treatment and more likely to report being unsatisfied when engaging with treatment [34, 36]. Overall, extant literature and current findings indicate an urgent need to better engage Black PWUD in substance use treatment. Potential strategies to do so include diversifying the addiction medicine and harm reduction workforce, ensuring equitable access to methadone vs. buprenorphine, and investing in evidence-based treatments for stimulant use disorder such as contingency management and medication assisted treatments [18, 35, 37, 38]. Policies that ensure insurance coverage of evidence-based treatments for stimulant use disorder may also be responsive. Finally, future research is needed to more deeply interrogate racial and ethnic equity in access to substance use treatment—including barriers to treatment and potential experiences of discrimination in care settings—among PWUD who have sought or who are indicated for substance use treatment [39, 40].

This study has several limitations. First, the measures were collected through self-report. As such, there is the risk of recall and social desirability bias. However, self-report measures of drug use behaviors are generally reliable and valid among PWUD [41]. Second, this study analyzes data from the RAPIDS baseline assessment, which was administered between August 2020 and February 2023. The current wave of the overdose crisis is characterized by a rapidly changing drug supply, and consequently, present findings may not reflect more emergent changes in the drug supply and drug use patterns, representing a limitation. Third, this study may be limited in its generalizability. The study stratified findings by Hispanic identity, but the RAPIDS study was limited to English speakers. Hispanic PWUD who spoke only Spanish were excluded from participation, potentially leading to underrepresentation of these individuals. Further research should explore whether there are differences in drug use patterns, harm reduction practices and access to treatment among those who speak Spanish. Finally, given that the objective of this analysis was to present stratified descriptive statistics to explore subgroup differences, all of our analyses are presented without statistical adjustment. Future research should consider multivariable analyses that more robustly consider the potential role of confounders, colliders, and mediators in the relationships among these variables of interest.

## Conclusions

In this cohort of PWUD in Rhode Island, we found differences in drug use patterns and overdose response experience by race and ethnicity. Black PWUD had significantly less regular use of unregulated opioids and less experience administering naloxone or performing rescue breathing. We also found that Black PWUD had a significantly less lifetime and current engagement in SUD treatment. Our findings suggest that increased investment in overdose education and naloxone distribution, the implementation of OPCs with supervised inhalation services, and evidence-based treatment for stimulant use disorder is warranted—particularly for communities of color. Additionally, further examination of barriers and facilitators to treatment for Black PWUD is needed to facilitate an equitable approach to the overdose crisis.

## Data Availability

Data and survey instrument are available upon request.
